# Construction of a prognostic model of colon cancer patients based on metabolism-related lncRNAs

**DOI:** 10.3389/fonc.2022.944476

**Published:** 2022-09-29

**Authors:** Chenyang Li, Qian Liu, Yiran Song, Wenxin Wang, Xiaolan Zhang

**Affiliations:** The Department of Gastroenterology and Hepatology, The Second Hospital of Hebei Medical University, Shijiazhuang, China

**Keywords:** colon cancer, metabolism, lncNRA, LASSO, prognostic model

## Abstract

**Background:**

Many studies have shown that metabolism-related lncRNAs may play an important role in the pathogenesis of colon cancer. In this study, a prognostic model for colon cancer patients was constructed based on metabolism-related lncRNAs.

**Methods:**

Both transcriptome data and clinical data of colon cancer patients were downloaded from the TCGA database, and metabolism-related genes were downloaded from the GSEA database. Through differential expression analysis and Pearson correlation analysis, long non-coding RNAs (lncRNAs) related to colon cancer metabolism were obtained. CRC patients were divided into training set and verification set at the ratio of 2:1. Based on the training set, univariate Cox regression analysis was utilized to determine the prognostic differential expression of metabolic-related lncRNAs. The Optimal lncRNAs were obtain by Lasso regression analysis, and a risk model was built to predict the prognosis of CRC patients. Meanwhile, patients were divided into high-risk and low-risk groups and a survival curve was drawn accordingly to determine whether the survival rate differs between the two groups. At the same time, subgroup analysis evaluated the predictive performance of the model. We combined clinical indicators with independent prognostic significance and risk scores to construct a nomogram. C index and the calibration curve, DCA clinical decision curve and ROC curve were obtained as well. The above results were all verified using the validation set. Finally, based on the CIBERSORT analysis method, the correlation between lncRNAs and 22 tumor-infiltrated lymphocytes was explored.

**Results:**

By difference analysis, 2491 differential lncRNAs were obtained, of which 226 were metabolic-related lncRNAs. Based on Cox regression analysis and Lasso results, a multi-factor prognostic risk prediction model with 13 lncRNAs was constructed. Survival curve results suggested that patients with high scores and have a poorer prognosis than patients with low scores (P<0.05). The area under the ROC curve (AUC) for the 3-year survival and 5-year survival were 0.768 and 0.735, respectively. Cox regression analysis showed that age, distant metastasis and risk scores can be used as independent prognostic factors. Then, a nomogram including age, distant metastasis and risk scores was built. The C index was 0.743, and the ROC curve was drawn to obtain the AUC of the 3-year survival and the 5-year survival, which were 0.802 and 0.832, respectively. The above results indicated that the nomogram has a good predictive effect. Enrichment analysis of KEGG pathway revealed that differential lncRNAs may be related to chemokines, amino acid and sugar metabolism, NOD-like receptor and Toll-like receptor activation as well as other pathways. Finally, the analysis results based on the CIBERSORT algorithm showed that the lncRNAs used to construct the model had a strong polarized correlation with B cells, CD8+T cells and M0 macrophages.

**Conclusion:**

13 metabolic-related lncRNAs affecting the prognosis of CRC were screened by bioinformatics methods, and a prognostic risk model was constructed, laying a solid foundation for the research of metabolic-related lncRNAs in CRC.

## Background

Clorectal cancer (CRC) is one of the most common gastrointestinal malignancies in the world, whose morbidity and mortality are increasing year by year. In 2018, China’s CRC accounted for the third in the national incidence of malignant tumors and the second in mortality, and the number of new cases and deaths was as high as 376,000 and 191,000, respectively (1). Genetics, living environment, diet and other factors are all risk factors resulting in CRC (2, 3). At present, with the development of medical diagnosis and treatment technology, tumor molecular target and biological therapy were set as the entry point, searching for target proteins and regulatory signal pathways related to tumor occurrence and development, so as to achieve better effects.

Tumor metabolism plays a key role in the occurrence and development of tumors, including sugar metabolism, nucleic acid metabolism, enzyme metabolism, and protein metabolism (4–7). Studies have shown that tumor metabolism has a significant correlation with tumor prognosis. Long non-coding RNAs (lncRNAs) are RNAs with a transcription length between 200 and 100 000 nt, which do not encode proteins themselves but participate in many physiological processes. Recent studies have shown that metabolism-related lncRNAs are related to the prognosis of a variety of cancers, including breast cancer and lung cancer (8, 9). However, the role of metabolism and related lncRNAs in CRC still remains unclear.

The study used bioinformatics methods to analyze the correlation between differential lncRNAs and metabolic genes, and obtain differentially expressed metabolic-related lncRNAs. Furthermore, we used LASSO regression analysis to construct a risk model to obtain metabolic-related lncRNAs that affect the prognosis of CRC, used single and multi-factor Cox analysis to construct a prognostic risk prediction nomogram model, and evaluated the correlation between genes and clinicopathological characteristicsin the model, providing a new idea for the prognosis of CRC.

## Materials and methods

### Data resources

The colon cancer-related data and metabolism-related data in this study were from the Cancer Genome Atlas (TCGA) (https://cancergenome.nih.gov) database and the Gene Set Enrichment Analysis database (GSEA, https://www.gsea-msigdb.org/gsea/index.jsp), respectively.

### Differential expression analysis

Limma package in the R software was used to perform differential analysis, and the differentially expressed metabolism-related genes and lncRNA in colon cancer tissues and adjacent tissues were explored in TCGA, with | log2FC |>1 and the false discovery rate was set as FDR< 0.05 for screening criteria. Pearson correlation analysis was used to calculate the correlation coefficient (R2) between lncRNAs and metabolism-related genes. The lncRNAs with R2>0.5 and p<0.05 were defined as metabolic-related lncRNAs.

### Construction of the prognostic model

The colon cancer patients were divided into the training set and validation set at the ratio of 2:1. Based on the training set, the single-factor Cox regression analysis of the metabolic-related lncRNAs was performed to obtain the lncRNAs related to the overall survival. P<0.05 was considered as statistically significant and was included in the LASSO regression analysis to determine the closely related lncRNAs. Multi-factor Cox regression generated risk coefficients and a risk regression model was then constructed. The risk scores of different patients were calculated. The prognostic risk score formula was constructed as follows: Risk score $\overset{n}{\underset{1}{\sum }}coefficients*Expression\;of\;metabolic-related\;lncRNAs\left(i\right)$ =. According to the median value of the risk score., the patients were divided into high-risk and low-risk groups, and the survival curve was used to compare the prognosis of the two groups for verification. The prediction effect of the area under the curve (AUC) evaluation model was analyzed by ROC curve analysis, all the above analyses were verified in the verification set.

### Clinical subgroup analysis

The survival package in R was applied for the subgroup analysis of lncRNAs risk model combined with the clinical subgroup characteristics of patients, such as age, gender, TNM staging. The ability of the risk model to distinguish high and low risk patients in different subgroups was clarified.

### Construction and evaluation of the nomogram

Univariate and multivariate Cox regression analysis were used to analyze risk scores and clinical factors including age, gender, tumor stage and TNM stage and screened independent prognostic factors. We built a nomogram based on the results of multivariate Cox regression including risk scores. The 3-year and 5-year OS for each patient were predicted based on the nomogram. At the same time, the C index, calibration curve and ROC curve were generated to evaluate the prediction effect of the model. The above results are all verified in the validation set to verify the stability of the results.

### GSEA enrichment analysis

KEGG analysis was performed on the selected metabolic-related prognostic lncRNAs based on the clusterProfiler package in R using GSEA software to identify genes with rich function and classify gene clusters (P<0.05).

### Correlation analysis between prognostic lncRNAs and tumor immunity

CIBERSORT is a tool for deconvolving the expression matrix of human immune cell subtypes based on the principle of linear support vector regression. The CIBERSORT analysis method (10, 11) was used to clarify the level of infiltration of 22 immune cells in CRC patients, then based on correlation analysis, the correlation of 13 prognostic metabolism-related lncRNAs with immune cell infiltration was determined, and the mechanism of prognostic lncRNAs affecting the progression of CRC was explored.

### CRC tissue sample collection

A total of 12 pairs of CRC tissues and noncancerous adjacent tissues were collected from patients who had undergone surgical resection at the Second Hospital of Hebei Medical University (Shijiazhuang, China). All patients had signed informed consent. This study was approved by the Ethical Review Committee of the Second Hospital of Hebei Medical University and was conducted in accordance with accepted ethical guidelines.

### RNA extraction and qRT-PCR analysis

Total tissue RNA was extracted using Trizol reagent (Vazyme, Nanjing, China) following the manufacturer’s protocols. Then, RNA samples were reverse transcribed by Hiscript III Reverse Transcriptase kit (Vazyme, Nanjing, China) and corresponding RNA expression was evaluated by qRT-PCR with ChamQ™ Universal SYBR qPCR Master Mix kit (Vazyme, Nanjing, China). GAPDH acted as the internal reference for normalization.

### Statistical analysis

The analysis in this study was completed by R software version 3.6.2, in which the limma package was used for differential gene acquisition, cluster Profiler and org.Hs.eg.db package were used for functional enrichment analysis, and survival package was used to perform Kaplan-Meie survival analysis.

## Results

### Differential expression analysis and correlation analysis to obtain metabolic-related lncRNAs

**Figure 1:**
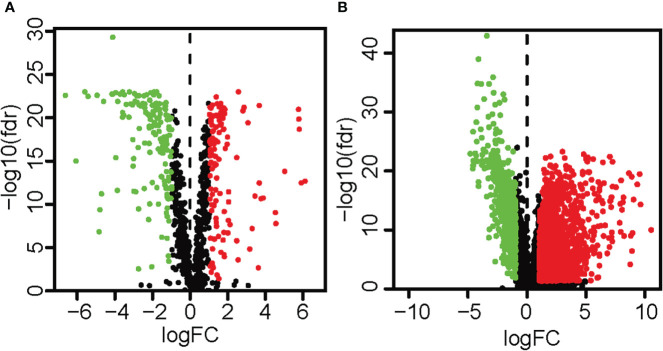
The volcano map highlighted the differentially expressed metabolism-related genes and lncRNAs in colon cancer tissues and surrounding tissues. Red showed a considerable up-regulation, green represented a significant down-regulation, and black represented no significant variation in gene expression. **(A)** The volcano map and heat map demonstrated the differentially expressed metabolism-related genes (| log2FC |>1, FDR< 0.05). **(B)** The volcano map and heat map demonstrated differentially expressed lncRNAs (| log2FC |>1, FDR< 0.05).

The expression data of 473 colon cancer tissues and 41 normal colon tissues was downloaded from the TGCA database, and the metabolism-related genes were obtained from the FerrDb database. A total of 279 differentially expressed metabolism-related genes were obtained, of which 151 genes were down-regulated and 128 genes were up-regulated (**Figure 1**; **Supplementary Materials Table 1**). In addition, a total of 2491 differentially expressed lncRNAs were identified, of which 675 lncRNAs were down-regulated and 1816 lncRNAs were up-regulated (**Figure 1**; **Supplementary Materials Table 2**). In addition, according to Pearson correlation analysis, 226 metabolic-related lncRNAs were obtained, and the standards were set as |R2|>0.5 and p<0.05 (**Supplementary Materials Table 3**).

### Construction of prognostic risk prediction model

**Figure 2:**
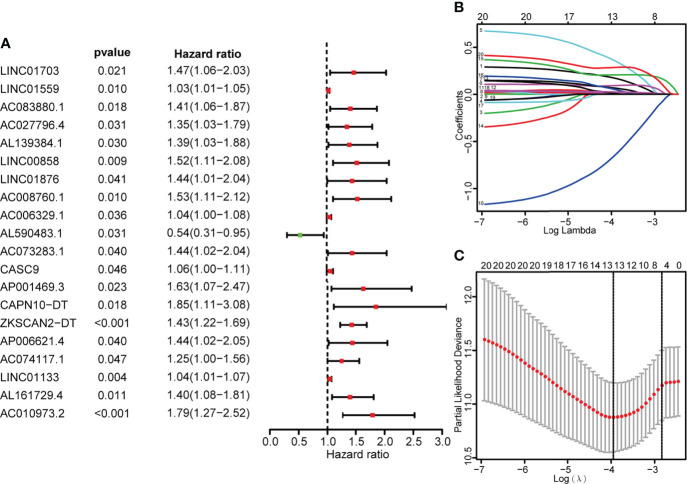
Identify metabolic-related lncRNAs having prognostic significance for colon cancer. **(A)** COX forest plot demonstrated that 20 metabolic-related lncRNAs were substantially correlated with the OS of colon cancer (P< 0.05). **(B, C)** The adjustment parameters of the LASSO regression model, LASSO coefficient spectrum of prognostic-related lncRNAs, 10-fold cross-validation to filtrate candidate necroptosisrelated lncRNAs in LASSO regression analysis.

**Figure 3:**
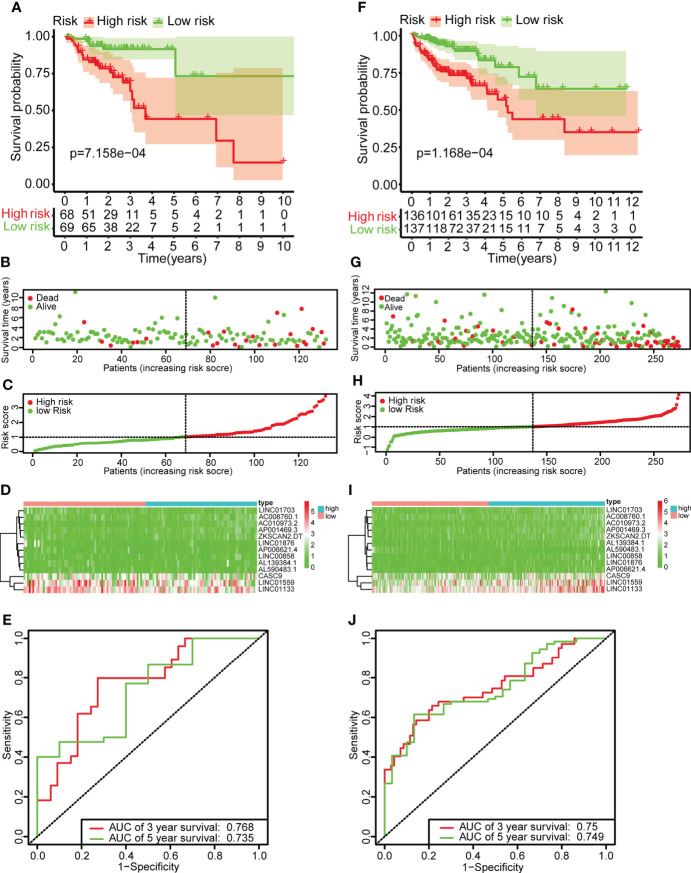
Construction of the risk scoring model. **(A, F)** Kaplan-survival Meier’s study of colon cancer patients in the training set and validation set indicated that the prognosis of the high-risk group was considerably poorer, while the low-risk group was better. **(B, G)** The survival rate and survival status of colon cancer patients in the training set and validation set. **(C, H)** The distribution of risk scores of 13 lncRNAs for colon cancer patients in the training and validation set. **(D, I)** Heat maps of 13 lncRNAs in the low-risk and high-risk group in the training set and validation set. **(E, J)** ROC curve analysis of colon cancer patients in the training set and validation set. The AUC values of the 3-year and 5-year survival rates of the training set were 0.768 and 0.735, respectively. The AUC values of the 3-year and 5-year survival rates of the validation set were 0.768 and 0.735, respectively.

Single-factor Cox regression analysis was performed on the selected metabolic-related lncRNAs, and a total of 20 lncRNAs related to prognosis were found. The 20 lncRNAs were included in the LASSO regression analysis to obtain the 13 most critical metabolic-related lncRNAs (**Figure 2**), and the multi-factor COX regression analysis was used to construct a prognostic risk prediction model based on the 13 metabolic-related lncRNAs (risk score $\overset{i}{\underset{1}{\sum }}\left(coef*exp\right)$ =)(**Supplementary Materials Table 4**). According to the median risk score, patients were divided into high-risk groups (n=136) and low-risk groups (n=137). The survival curve showed that the OS of the low-risk group was significantly higher than that of the high-risk group (p<0.001, **Figure 3**). The scatter plot and risk curve revealed that compared with the high-risk group, the low-risk group has lower risk factors and mortality (**Figure 3B****)**. The heat map showed the expression levels of 13 lncRNAs between the high-risk group and the low-risk group (**Figure 3**). The AUC of the ROC curve for 3-year and 5-year survival were 0.768 and 0.735, respectively (**Figure 3**). Similar results were obtained in the validation set (**Figure 3J**).

### Subgroup survival analysis

**Figure 4:**
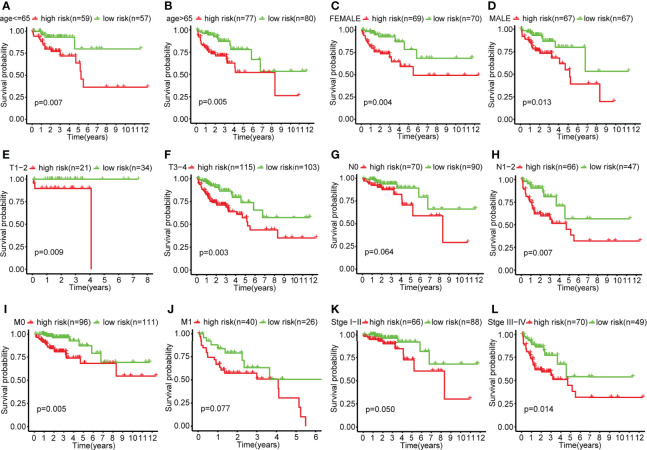
Survival curves in each clinical subgroup of the high-risk and low-risk group of colon cancer patients. **(A)** Survival curves of the high-risk and low-risk groups in the age ≤ 65 subgroup. **(B)** Survival curves of the high-risk and low-risk groups in the age>65 subgroup. **(C)** Survival curves of the high-risk and low-risk groups in the female subgroup. **(D)** Survival curves of the high-risk and low-risk groups in the male subgroup. **(E)** Survival curves of the high-risk and low-risk groups in the T1-2 subgroup. **(F)** Survival curves of the high-risk and low-risk groups in the T3-4 subgroup. **(G)** Survival curves of the high-risk and low-risk groups in the N0 subgroup. **(H)** Survival curves of the high-risk and low-risk groups in the N1-2 subgroup. **(I)** Survival curves of the high-risk and low-risk groups in the M0 subgroup. **(J)** Survival curves of the high-risk and low-risk groups in the M1 subgroup. **(K)** Survival curves of the high-risk and low-risk groups in the stageI-II subgroup. **(L)** Survival curves of the high-risk and low-risk groups in the stageIII-IV subgroup.

The results of subgroup analysis showed that the risk score model in the subgroups of age greater than 65, younger than 65, female, male, T1-2, T3-4, N1-2, M0, and stage III-IV, the OS of the low-risk group was significantly higher than that of the high-risk group, and the difference was statistically significant (P<0.05). However, there was no statistical difference in OS between the high-risk and low-risk groups in the N0, M1, and stage I-II-subgroups (**Figure 4**).

### Clarify independent prognostic factors

**Figure 5:**
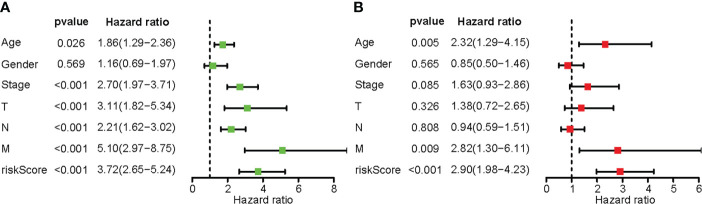
The predictive usefulness of clinicopathological features and risk ratings. **(A)** Univariate Cox regression of colon cancer patients. **(B)** Multivariate Cox regression of colon cancer patients.

Univariate Cox regression analysis showed that age, TNM stage, and risk score were related to the patient’s prognosis (P<0.05) (**Figure 5**). However, results of multivariate Cox regression showed that only age, M staging and risk score were closely related to the prognosis of CRC patients and were independent prognostic factors (**Figure 5**).

### Construction of the Nomogram

**Figure 6:**
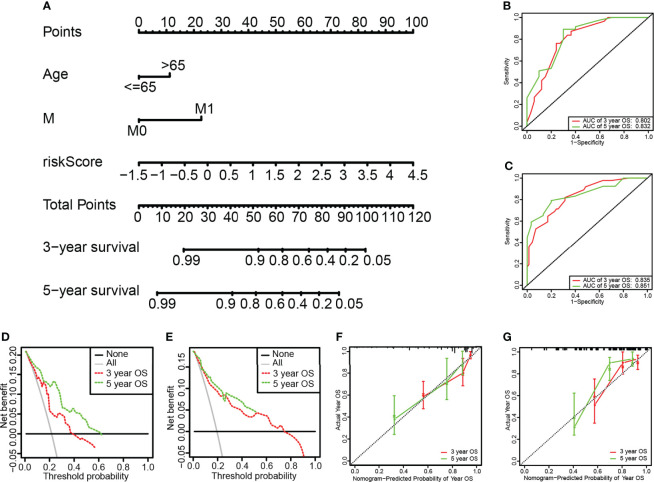
Construction and assessment of the Nomogram. **(A)** The nomogram for predicting the 3-year and 5-year survival rate of the training set and the validation set. **(B, C)** ROC curve of the training set and validation set. The AUC values of the 3-year and 5-year survival rates of the training set were 0.802 and 0.832, respectively. The AUC values of the 3-year and 5-year survival rates of the validation set were 0.835 and 0.851, respectively. **(D, E)** Calibration chart nomogram of the training set and validation set. **(F, G)** DCA clinical decision curve of training set and validation set.

The Nomogram was constructed based on independent prognostic factors determined by the multivariate Cox regression results, such as age, M, and risk score (**Figure 6**). The C index of the nomogram in the training set were 0.823, the AUC of the 3-year and 5-year OS were 0.802 and 0.832, respectively (**Figure 6**). The results of the calibration curve (**Figure 6**), and the clinical decision curve (**Figure 6**) showed the model had good predictive performance and can bring benefits to patients. The above results were all verified in the internal validation set to verify the stability of the results (**Figures 6C, E****)**.

### KEGG pathway enrichment analysis

**Figure 7:**
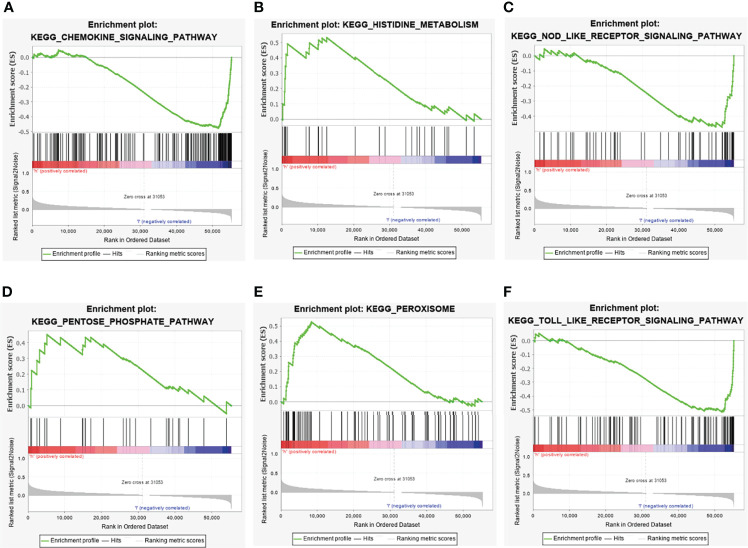
GSEA pathway enrichment analysis of metabolism-related lncRNAs. **(A)** Chemokine signaling pathway. **(B)** Histidine metabolism. **(C)** NOD- like receptor signaling pathway. **(D)** Pentose phosphate pathway. **(E)** Peroxisome. **(F)** Toll like receptor signaling pathway.

The KEGG pathway enrichment analysis of the differentially expressed metabolic-related lncRNAs showed that differential lncRNAs were mainly related to the following pathways: chemokine pathways, amino acid and sugar metabolism, NOD-like receptors and Toll-like receptor activation (**Figure 7**).

### Correlation analysis between prognostic lncRNAs and immune cell infiltration

**Figure 8:**
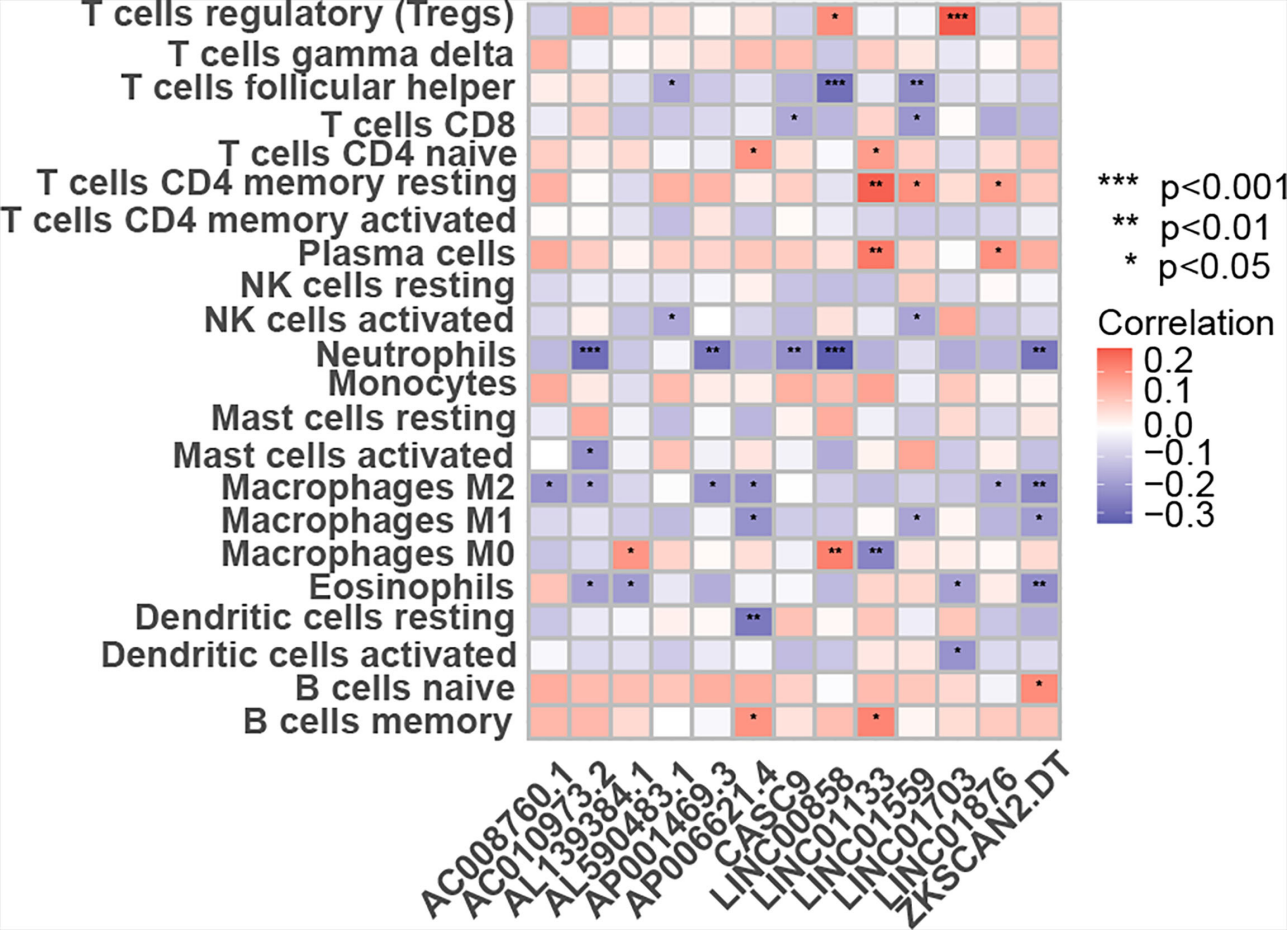
The link between the percentage of immune cells based on the CIBERSORT and the expression of 13 lncRNAs for creating the risk-scoring model. *p < 0.05, **p < 0.01, ***p < 0.001.

The CIBERSORT-based analysis method clarified the infiltration level of 22 immune cells in colon cancer patients, and the results showed that lncRNAs used to construct the model had a strong correlation with the polarization of B cells, CD8+ T cells and M0 macrophages (**Figure 8**), indicating that these lncRNAs may regulate tumor growth and progression by affecting the tumor immune infiltration microenvironment.

### Validation of metabolic-related lncRNAs expression in tissue samples

**Figure 9:**
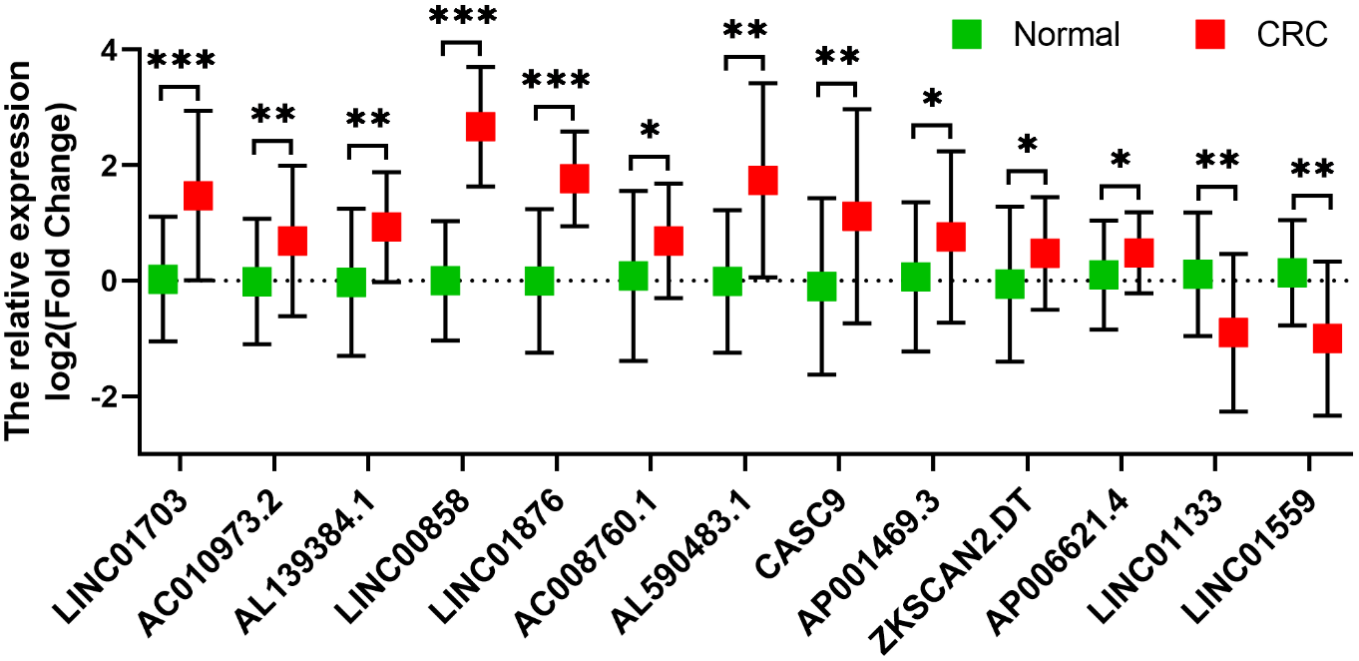
Validation of the expression of the selected metabolism-related lncRNAs in 12 pairs of clinical tissues (n=12). The expression levels of LINC01703, AL139384.1, LINC00858, LINC01876, AC008760.1, AL590483.1, CASC9, AP001469.3, ZKSCAN2.DT, AP006621.4 and AC010973.2 were significantly higher in CRC tissue samples, while LINC01559 and LINC01133 were significantly lower in CRC tissue samples. *p < 0.05, **p < 0.01, ***p < 0.001.

The expression levels of selected metabolic-related lncRNAs were further evaluated and validated in tissues. As illustrated in **Figure 9**, the expression levels of LINC01703, AL139384.1, LINC00858, LINC01876, AC008760.1, AL590483.1, CASC9, AP001469.3, ZKSCAN2.DT, AP006621.4 and AC010973.2 were significantly higher in CRC tissue samples, while LINC01559 and LINC01133 were significantly lower in CRC tissue samples. The detailed sequence of primers used were listed in **Supplementary Table 5**. These results further confirmed the correctness of the above bioinformatics analyses.

## Discussion

How to predict the prognosis of colon cancer is an urgent problem for gastrointestinal surgeons. With the development of clinical diagnosis and treatment, some prognostic factors have been discovered, including tumor size, tumor grade and stage. High-throughput biological technology has been widely used to predict cancer recurrence and tumor metastasis by detecting lncRNA or gene changes. In recent years, many studies have shown that changes in the expression patterns of metabolism-related genes are closely related to the occurrence and prognosis of colon cancer [13]. In-depth study of metabolism-related lncRNAs is expected to provide guidance for clinical decision-making.

Colon cancer is a common malignant tumor in the digestive system, which seriously affects patients’ survival (12, 13). How to predict the prognosis of colon cancer is an urgent problem for gastrointestinal surgeons. With the development of clinical diagnosis and treatment, some prognostic factors have been discovered, including tumor size, tumor grade and stage. High-throughput biological technology has been widely used to predict cancer and tumor metastasis by detecting lncRNAs or gene changes. In recent years, many studies have shown that changes in the expression patterns of metabolism-related genes are closely related to the occurrence and prognosis of colon cancer (14). To study metabolism-related lncRNAs in depth is expected to provide guidance for clinical decision-making.

This study identified 13 metabolic lncRNAs related to the prognosis of CRC patients through bioinformatics methods, namely LINC01703, LINC01559, AC083880.1, AC027796.4, AL139384.1, LINC00858, LINC01876, AC008760.1, AC006329.1 AL590483.1, AC073283.1, CASC9, AP001469.3, CAPN10-DT, ZKSCAN2-DT, AP006621.4, AC074117.1, LINC01133, AL161729.4 and AC010973.2, thereby building a risk model for predicting prognosis. Combined with clinicopathological analysis, this model can be used as an independent predictor for the prognosis of lung cancer.

Among them, LINC01703 has been reported to be of great significance in the diagnosis of lung adenocarcinoma. Wang et al. found that LINC01703 enhanced the invasiveness of NSCLC cells by changing miR-605-3p/MACC1 (15). In addition, the bioinformatics analysis of the sequencing data of lung adenocarcinoma showed that LINC01703 and AC074117.1 can be used as potential diagnostic biomarkers for lung adenocarcinoma (16, 17). LINC01559 played an important role in gastrointestinal tumors such as pancreatic cancer, gastric cancer and liver cancer (18–21). AC083880.1 was also an important lncRNA, which was related to the activation of autophagy-related pathways in ovarian cancer (22). AC027796.4 was regarded as a potential biomarker for prostate cancer (23). As a ceRNA, LINC01133 regulated APC expression and Wnt/β-catenin pathway by releasing miR-106a-3p to inhibit gastric cancer progression (23). LINC00858 regulated PAK2 signaling pathway through sponge adsorption of miR-4766-5p to promote the progression of colorectal cancer (24). Studies have confirmed that AC008760.1, AC006329.1, AL590483.1, CASC9 and AC010973.2 (25) can be potential biomarkers for colorectal cancer (25–28), while there are few studies about AL139384.1, LINC01876, AP001469.3, CAPN10-DT, ZKSCAN2-DT, AP006621.4, and AL161729.4 in solid tumors.

Pathway enrichment analysis revealed that metabolism-related lncRNAs were mainly related to pathways such as chemokine pathways, metabolic collaterals, NOD-like receptors and Toll-like receptor activation. Liu et al. (29) found that the activity of gastric cancer cells decreased after lncRNA and UCA-1 were knocked out, in which the chemokine pathway played an important role. Besides, Satoh et al. (30) found that LINC01876 can up-regulate chemokine (C-C motif) ligand 2, and promoted the proliferation of macrophages and myeloid-derived suppressor cells in hepatocellular carcinoma cell lines. While in colorectal cancer, CASC9 interacted with CPSF3 to regulate TGF-β signal transduction (31). LINC01133 inhibited epithelial-mesenchymal transition and metastasis of colorectal cancer by interacting with SRSF6 LINC01133 (32). At present, there are few research on signal pathways related to amino acid and sugar metabolism.

A large number of studies have shown that there is a significant correlation between tumor cell metabolism and immune cell infiltration in tumor tissues (33–35). In order to further reveal the role of 13 lncRNAs in colon cancer, we analyzed the correlation between 13 prognostic-related lncRNAs in the expression matrix of the colon cancer and 22 kinds of immune cell infiltration based on the CIBERSORT algorithm, whose results showed that these 13 lncRNAs showed a strong correlation with neutrophils, follicular helper T cells and polarized M2 macrophages, suggesting that they may pose impact on the prognosis of colon cancer by affecting the tumor immune microenvironment. The specific regulatory mechanism remains to be studied in depth.

## Conclusions

The relationship between metabolic-related lncRNAs and the prognosis of CRC was preliminarily explored through bioinformatics methods, a model based on 13 metabolic-related lncRNAs was established to predict the prognosis of CRC, and the stability of the model in multiple data sets was fully verified. It provided a new direction for the study of metabolism and colon cancer. We still need further *in vitro* and *in vivo* studies to clarify the role and specific mechanisms of metabolism-related lncRNAs in the occurrence and development of colon cancer.

## Data availability statement

The original contributions presented in the study are included in the article/**Supplementary Material**. Further inquiries can be directed to the corresponding author.

## Author contributions

XZ designed the experiments. CL performed the analysis. CL and QL analyzed the TCGA data. CL, YS and WW wrote and reviewed the manuscript. All authors contributed to the article and approved the submitted version.

## Acknowledgments

We would like to acknowledge TCGA for free use.

## Conflict of interest

The authors declare that the research was conducted in the absence of any commercial or financial relationships that could be construed as a potential conflict of interest.

## Publisher’s note

All claims expressed in this article are solely those of the authors and do not necessarily represent those of their affiliated organizations, or those of the publisher, the editors and the reviewers. Any product that may be evaluated in this article, or claim that may be made by its manufacturer, is not guaranteed or endorsed by the publisher.

## Supplementary material

Supplementary Table 1 A total of 279 differentially expressed metabolism-related genes were obtained, of which 151 genes were down-regulated and 128 genes were up-regulated.

Supplementary Table 2 A total of 2491 differentially expressed lncRNAs were identified, of which 675 lncRNAs were down-regulated and 1816 lncRNAs were up-regulated.

Supplementary Table 3 According to Pearson correlation analysis, 226 metabolic-related lncRNAs were obtained, and the standards were set as |R2|>0.5 and p<0.05.

Supplementary Table 4 Multi-factor COX regression analysis was used to construct a prognostic risk prediction model based on the 13 metabolic-related lncRNAs.

Supplementary Table 5 Detailed sequences of primers used in qRT-PCR experiments.
